# Identification of a Novel Tumor Microenvironment Prognostic Signature for Bladder Urothelial Carcinoma

**DOI:** 10.3389/fonc.2022.818860

**Published:** 2022-03-01

**Authors:** Chaojie Xu, Dongchen Pei, Yi Liu, Yang Yu, Jinhua Guo, Nan Liu, Zhengjun Kang

**Affiliations:** Department of Urology, The Fifth Affiliated Hospital of Zhengzhou University, Zhengzhou University, Zhengzhou, China

**Keywords:** tumor microenvironment, prognosis, bladder urothelial carcinoma, immunotherapy, non-negative matrix factorization

## Abstract

**Background:**

The tumor microenvironment (TME) regulates the proliferation and metastasis of solid tumors and the effectiveness of immunotherapy against them. We investigated the prognostic role of TME-related genes based on transcriptomic data of bladder urothelial carcinoma (BLCA) and formulated a prediction model of TME-related signatures.

**Methods:**

Molecular subtypes were identified using the non-negative matrix factorization (NMF) algorithm based on TME-related genes from the TCGA database. TME-related genes with prognostic significance were screened with univariate Cox regression analysis and lasso regression. Nomogram was developed based on risk genes. Receiver operating characteristic (ROC) curve and decision curve analysis (DCA) were used for inner and outer validation of the model. Risk scores (RS) of patients were calculated and divided into high-risk group (HRG) and low-risk group (LRG) to compare the differences in clinical characteristics and PD-L1 treatment responsiveness between HRG and LRG.

**Results:**

We identified two molecular subtypes (C1 and C2) according to the NMF algorithm. There were significant differences in overall survival (OS) (p<0.05), progression-free survival (PFS) (p<0.05), and immune cell infiltration between the two subtypes. A total of eight TME-associated genes (*CABP4*, *ZNF432*, *BLOC1S3*, *CXCL11*, *ANO9*, *OAS1*, *FBN2*, *CEMIP*) with independent prognostic significance were screened to build prognostic risk models. Age (p<0.001), grade (p<0.001), and RS (p<0.001) were independent predictors of survival in BLCA patients. The developed RS nomogram was able to predict the prognosis of BLCA patients at 1, 3, and 5 years more potentially than the models of other investigators according to ROC and DCA. RS showed significantly higher values (p = 0.047) in patients with stable disease (SD)/progressive disease (PD) compared to patients with complete response (CR)/partial response (PR).

**Conclusions:**

We successfully clustered and constructed predictive models for TME-associated genes and helped guide immunotherapy strategies.

## Introduction

Bladder urothelial carcinoma (BLCA) is the pathological type of bladder cancer with the highest percentage, about 90% (1). There were globally approximately 550,000 new patients and 200,000 deaths in 2018 (2). Despite significant advances in the treatment of BLCA with radiotherapy, surgery, and targeted therapy, the prognosis of BCLA patients remains poor, with 30% of them having muscle-invasive bladder cancer (MIBC) at initial consultation (3). However, patients with MIBC are characterized by rapid disease progression and low survival, with a 5-year tumor-specific mortality rate of >50% (4). Therefore, a validated prognostic risk model can help guide individualized treatment of BLCA patients to improve their prognosis.

At present, the 8th edition of the TNM staging method published by the Union International Center of Cancer (UICC) is one of the most valuable indicators to determine the prognosis of patients with BLCA (5). However, due to the heterogeneity of BLCA, the prognosis of patients with the same TNM stage may vary considerably (6–8). In addition, multiple comprehensive BLCA molecular typing based on genetic analysis can forecast the overall survival (OS) of individuals, such as ferroptosis-associated gene signature (9), autophagy-associated gene signature (10), and RNA binding protein-associated gene signature (11). Therefore, we considered the use of gene signatures as biomarkers that could predict individual prognosis and drug responsiveness, thus improving clinical outcomes in BLCA patients.

The tumor microenvironment (TME) includes solid tumor cells, vascular network, extracellular matrix, secreted factors (cytokines, chemokines), and distantly recruited cells such as activated B cells and macrophages (12). Overall, this homeostatic system supports the progression and recurrence of malignancies and has important implications in chemoresistance and immunotherapy (13, 14). Mesenchymal cells such as fibroblasts within TME are associated with the T-cell efflux phenotype in bladder cancer (15). In addition, the non-immune cellular components of TME also influence the therapeutic response, for example, the secretion of TGF-β by fibroblasts can lead to efflux of immune cells or resistance to chemotherapeutic agents, and therefore the therapeutic effect of the tumor varies with the degree of stromal cell infiltration (16). Therefore, tumor tissue gene expression profiles can reflect the relationship between TME and patient prognosis.

In summary, TME-associated gene signature can enhance the reliability of forecasting patient prognosis. Therefore, we aimed to design a prediction model combining TME-related gene signatures and patient clinical characteristics and develop a nomogram to forecast the prognosis of BLCA patients at 1, 3, and 5 years.

## Materials and Methods

### Data Download and Preprocessing

We downloaded transcriptome data and clinical annotations from the Genomic Data Commons. The TCGA-BLCA (The Cancer Genome Atlas-Bladder Cancer) cohort consisted of 433 RNA sequencing samples, including 19 normal profiles and 414 tumor profiles. We removed samples without clinical follow-up information and microdissection from the TCGA-BLCA cohort, resulting in the inclusion of a total of 359 samples. We also downloaded the simple nucleotide variation data in the TCGA database for further analysis of copy number variation (CNV).

Moreover, for external validation, data for the cohort of GSE31684 were obtained from the GEO database. The microarray data of GSE31684 from Affymetrix Human Genome U133 Plus 2.0 Array, we downloaded the normalized matrix file directly. All dates contain survival information.

TME-related genes were obtained from published studies (17–23), and a total of 4061 genes were included (**Table S1**). We used the UCSC Xena (https://xenabrowser.net/) to download the TCGA-GDC pan-cancer data.

### Screening for TME-Related Differentially Expressed Genes

The differentially expressed TME-associated genes in BLCA tissues and adjacent tissues were screened, and the screened differential genes and their expression were organized into a gene expression matrix with a corrected p<0.05 and the absolute value of differential expression multiplicity >1 (FDR<0.05 and | log2Fold Change|>1) was set as the threshold value.

### Identification of Molecular Subtypes Using Non-Negative Matrix Factorization (NMF) Algorithm

Fifty iterations of the sample were performed using NMF for extracting biological correlation coefficients and predicting the inner feature structure in gene expression matrices (24). We observed performance for the number of clusters k between two and ten.

### Comparison of Immune Scores Between Clusters

Microenvironment Cell Populations (MCP) counters allow quantification of the absolute abundance of eight immune cells and two stromal cells from transcriptomic data (24). We evaluated infiltrating cell scores between clusters, which included neutrophils, NK cells, and myeloid dendritic cells, among others. Then we evaluated the infiltrating cells between clusters.

### Univariate Cox Regression Analysis and Lasso Regression Analysis

At the ratio of 7:3, 359 samples were divided into the training set and validation set with no one as the control. TME-related differentially expressed genes were subjected to univariate Cox analysis to get the prognostic genes. The lasso method prevents model overfitting by building a penalized feature to build a more refined model. We then applied lasso Cox regression to minimize the amounts of genes in the prognostic modeling. The results of the lasso regression analysis on the variables affecting the outcome of individuals with BLCA were incorporated into multivariate Cox regression analysis.

### Construction of a Nomogram Combined With Risk Score (RS) and Clinical Features

The TCGA cohort was used to build a nomogram to forecast the prognosis of individuals with BLCA, with variables including RS and clinical characteristics. RS was calculated according to the expression of differential genes and regression analysis coefficient values. The formula is shown below:


$$riskscore=\sum_{i=1}^{n}\left(coefi*Xi\right)$$

We classify the cases into high-risk group (HRG) and low-risk group (LRG) according to the median RS. The receiver operating characteristic (ROC) curve was utilized to assess the predictive value of RS for prognosis. Kaplan-Meier (K-M) curves were drawn and the Log-rank method was used to evaluate OS.

### Prediction Model Evaluation

ROC, calibration curve (bootstrap 1000), and decision curve analysis (DCA) were applied to assess the confidence validity of the model. Our RS nomogram was also compared with those of other established models. RMS time was used to assess the prognostic accuracy of the models beyond 60 months of patient survival.

### Gene Set Enrichment Analysis (GSEA)

The c2.cp.kegg.v7.4.symbols and c5.go.v7.4.symbols collection were used to explore the function annotation in HRG and LRG by GSEA software. Gene sets with FDR < 0.05 were was considered statistically significant.

### Immunotherapy Prediction

We selected patients with urologic tumors in the IMvigor210 cohort who had received programmed death-ligand 1 (PD-L1) blockade treatment to predict response to immunotherapy. This cohort had a total of 348 cases, containing 232 death samples and 116 censored, all of which contained survival data. BLCA patients who received anti-PD-L1 therapy could be classified into the following categories according to the patient’s response: complete response (CR), partial response (PR), stable disease (SD), and progressive disease (PD). Among them, CR and PR are recognized as patients who respond to immunotherapy. SD and PD are recognized as patients who do not respond to immunotherapy. We computed RS for each case and classified them as HRG and LRG based on the median value of RS.

### Statistical Analysis

Statistical analysis and graphical work were finished in the R environment (version 4.1.1). Volcano maps were drawn using the “ggplot2” package. Violin plots were drawn with “ggpubr” package. Cox regression analyses were performed by the “survival” package. We used the Chi-square test or Fisher’s exact test to measure the difference between training and validating sets and the relationship between clinical data and RS. K-M survival curves with log-rank tests were plotted by the “survminer” package. The ROC curves were depicted by the “timeROC” package. Calibration curves were derived from the “rms” package. The restricted mean survival (RMS) package was for computing the C-index for each of the models. p < 0.05 is considered to have statistical difference.

## Results

### Patient Characteristics and Analysis of Differentially Expressed Genes Associated With TME in BLAC Patients

**Figure 1:**
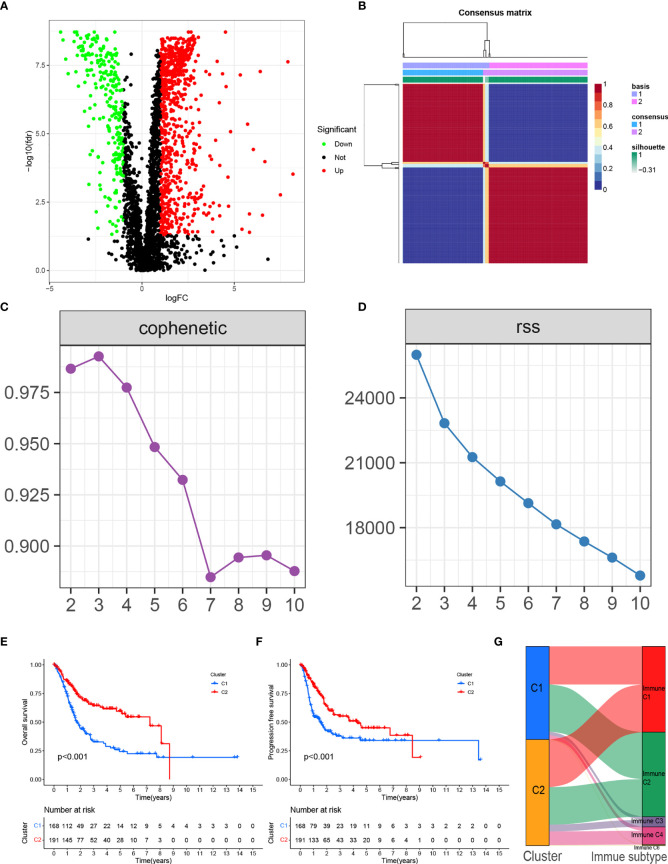
**(A)** Volcano plot of differentially expressed genes in BLCA from the TCGA database. **(B)** Consensus map clustered *via* the non-negative matrix factorization (NMF) algorithm. **(C)** The cophenetic correlation coefficient is used to reflect the stability of the cluster obtained from NMF. **(D)** RSS is used to reflect the clustering performance of the model. **(E)** Overall survival (OS) showed significant differences between C1 and C2. **(F)** Progression-free survival (PFS) showed significant differences between C1 and C2. **(G)** Alluvial plot showing the percentage of C1 and C2 between molecular subtypes.

The TCGA-BLCA cohort consisted of 433 RNA sequencing samples, including 19 normal samples and 414 tumor samples. The characteristics of the cases enrolled in this study were shown in **Table 1** after pre-processing. In total, there were 1014 TME-associated genes (FDR<0.05 and |log_2_FC|>1) differentiated in expression between BLCA patients and regular bladder tissue. The top 50 up-regulated and down-regulated genes with differential expression were plotted as volcanoes (**Figure 1A**).

**Table 1:** Clinicopathological characteristics of BLCA patients from the TCGA and GEO databases.

Characteristics	TCGA-BLCA cohort N = 359	GSE31684 N = 93
**Age**		
<=65	141 (39.28%)	28 (39.41%)
>65	218 (60.72%)	65 (60.59%)
**Gender**		
Female	99 (27.58%)	25 (26.88%)
Male	260 (72.42%)	68 (73.12%)
**Grade**		
High	342 (95.26%)	87 (93.50%)
Low	14 (3.9%)	6 (6.50%)
Unknow	3 (0.84%)	0 (0.00%)
**Stage**		
I-II	113 (31.48%)	74 (79.57%)
III-IV	244 (67.97%)	11 (11.83%)
Unknow	2 (0.56%)	8 (8.60%)
**T**		
T0-T2	107 (29.81%)	NA
T3-T4	223 (62.12%)	NA
Unknow	29 (8.08%)	NA
**M**		
M0	164 (45.68%)	NA
M1	11 (3.06%)	NA
Unknow		NA
**N**	184 (51.25%)	
N0-N1	251 (69.92%)	NA
N2-N3	72 (20.06%)	NA
Unknow	36 (10.03%)	NA
**Neoadjuvant therapy**		
Yes	10 (2.79%)	NA
No	349 (97.21%)	NA
**Radiotherapy**		
Yes	15 (4.18%)	NA
No	243 (67.69%)	NA
Unknow	101 (28.13%)	NA
**Survival status**		
Alive	201 (55.99%)	28 (30.11%)
Dead	158 (44.01%)	65 (69.89%)
**The median follow-up time** **(year)**	5.19	2.57

### Molecular Subtypes of TME-Related Genes According to the NMF Algorithm

**Figure 2:**
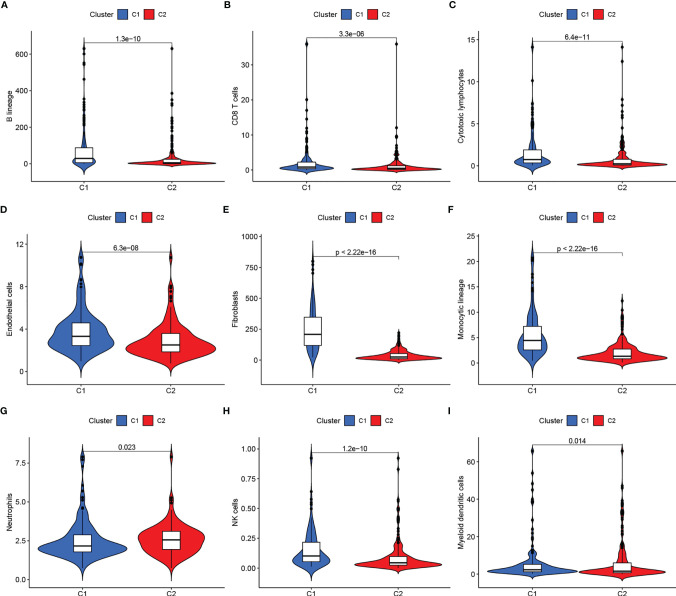
**(A–I)** Immune scores of cells of the tumor microenvironment (TME) showed significant differences.

We clustered genes based on the differential expression TME-related genes by the NMF algorithm. When k=2, C1 and C2 were formed based on covariance and RSS (**Figures 1B–D**). Survival results showed that cases in the C1 cluster had better OS and PFS than those in the C2 cluster (**Figures 1E, F**). There were differences in immune scores between C1 and C2 for nine cell types, including CD8+ T cells (p<0.05), cytotoxic lymphocytes (p<0.05), B cells (p<0.05), neutrophils (p = 0.023), monocytes (p<0.05), endothelial cells (p<0.05), fibroblasts (p<0.05), NK cells (p<0.05), and myeloid dendritic cells (p = 0.014) (**Figures 2A–I**). The international transcriptomic immune typology of solid tumors has established six immune subtypes, including wound healing (Immune C1), IFN-gamma dominant (Immune C2), inflammatory (Immune C3), lymphocyte depleted (Immune C4), immunologically quiet (Immune C5), and TGF-beta dominant (Immune C6). Our molecular subtyping results were compared with the former and the results are shown in **Figure 1G**.

### Developing an RS Prediction Model for TME-Related Genes

**Figure 3:**
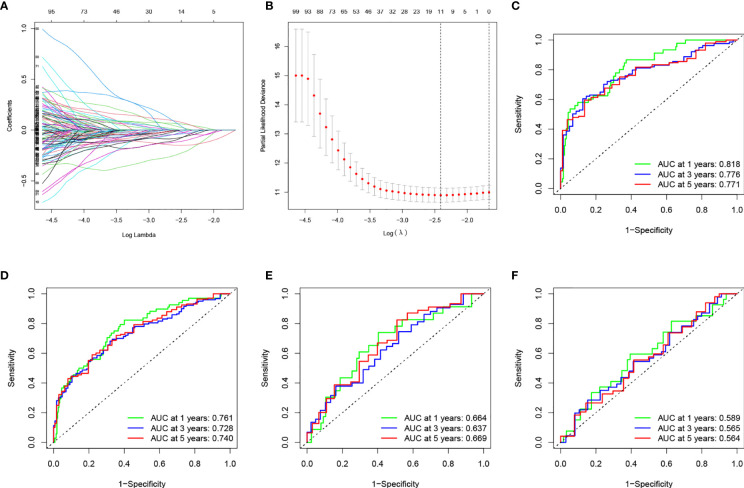
**(A)** Changing the trajectory of each independent variable (the abscissa represents the corrected lambda and the ordinate represents the coefficient of the independent variable). **(B)** log value of the independent variable lambda (the abscissa represents the CI of each lambda, and the ordinate represents errors in cross-validation). **(C–F)** Construction and validation of the TME-related eight-gene risk score (RS) for BLCA with 1-, 3- and 5-year receiver operating characteristic (ROC) within different cohorts: **(C)** TCGA training cohort; **(D)** entire TCGA cohort; **(E)** TCGA testing cohort; **(F)** GEO cohort.

At the ratio of 7:3, 359 samples were randomly divided into a training set (n = 252) and validation set (n = 107) (**Table 2**). Baseline features of the patients demonstrated no obvious distinction between them in terms of gender, age, pathological grade, treatment history, and TNM stage (p>0.05). Univariate Cox analysis was performed on the training cohort with 173 differentially TME-associated genes (p < 0.05) (**Table S2**). Lasso regression was applied to achieve reduction in the number of genes while remaining highly accurate. The traces of independent variables showed a gradual increase in the number of independent coefficients that converge to zero as λ decreases (**Figure 3A**). We selected 11 genes as candidate genes according to the best value of λ (**Figure 3B**). Then TME-related genes with significant differences were screened according to a multifactorial Cox proportional risk model. Finally, eight genes were obtained, constructing the following equation:


$$\begin{array}{c} risk\;score\;(RS)=-(0.2823\times CABP4)-(0.2536\times ZNF432)\\ -(0.4745\times BLOC1S3)-(0.1731\times CXCL11)\\ -(0.2813\times AN09)-(0.2149\times OAS1)\\ +(0.1846\times FBN2)+(0.2785\times CEMIP) \end{array}$$

**Table 2:** Comparison of TCGA training and testing cohort.

Characteristics	TCGA Testing Cohort N = 107	TCGA Training Cohort N = 252	*p*-Value
**Age**			0.5587
<=65	45 (42.06%)	96 (38.1%)	
>65	62 (57.94%)	156 (61.9%)	
**Gender**			0.1973
Female	35 (32.71%)	64 (25.4%)	
Male	72 (67.29%)	188 (74.6%)	
**Grade**			1
High	103 (96.26%)	239 (94.84%)	
Low	4 (3.74%)	10 (3.97%)	
Unknow	0 (0%)	3 (1.19%)	
**Stage**			0.5564
I-II	31 (28.97%)	82 (32.54%)	
III-IV	76 (71.03%)	168 (66.67%)	
Unknow	0 (0%)	2 (0.79%)	
**T**			0.806
T0-T2	30 (28.04%)	77 (30.56%)	
T3-T4	67 (62.62%)	156 (61.9%)	
Unknow	10 (9.35%)	19 (7.54%)	
**M**			1
M0	46 (42.99%)	118 (46.83%)	
M1	3 (2.8%)	8 (3.17%)	
Unknow	58 (54.21%)	126 (50%)	
**N**			0.254
N0-N1	79 (73.83%)	172 (68.25%)	
N2-N3	17 (15.89%)	55 (21.83%)	
Unknow	11 (10.28%)	25 (9.92%)	
**Neoadjuvant therapy**			0.7362
Yes	2 (1.87%)	8 (3.17%)	
No	105 (98.13%)	244 (96.83%)	
**Radiotherapy**			0.9045
Yes	4 (3.74%)	11 (4.37%)	
No	77 (71.96%)	166 (65.87%)	
Unknow	26 (24.3%)	75 (29.76%)	

**Figure 4:**
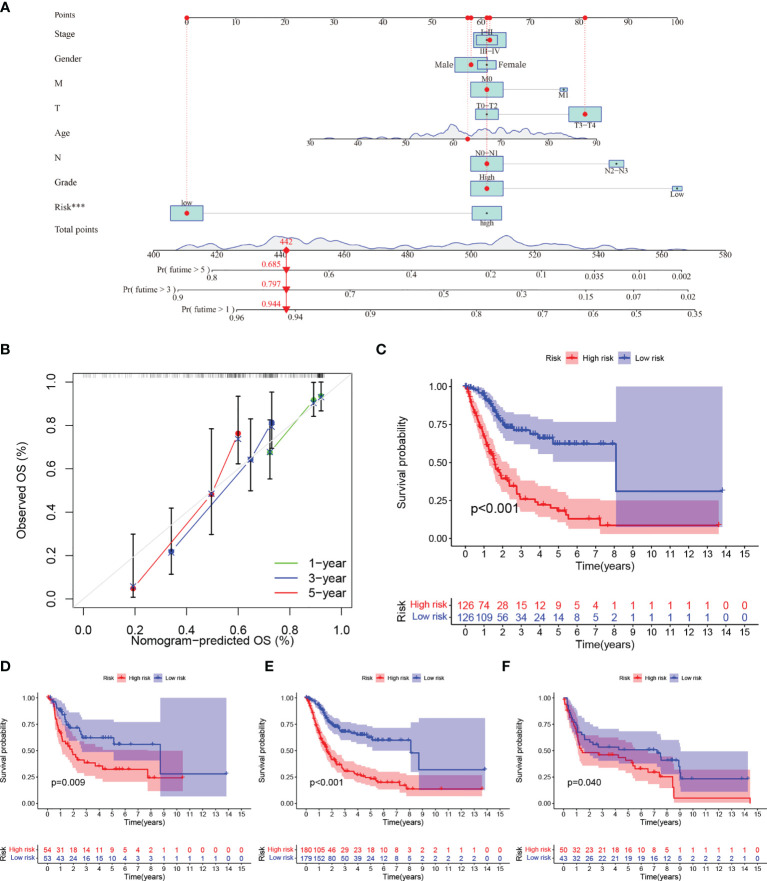
**(A–C) (A)** Nomogram predicting the 1-, 3- and 5-year OS for patients. The points identified on the point scale of each variable are totaled. Finally, beneath the total points, the probability of 1-, 3- or 5-year survival is projected on the scales below. **(B)** Calibration curves for the nomogram predicted 1-, 3- and 5-year OS for patients in relation to actual survival. **(C–F)** Construction and validation of the TME-related eight-gene risk score (RS) for BLCA with Kaplan–Meier (KM) curves within different cohorts: **(C)** TCGA training cohort; **(D)** TCGA testing cohort; **(E)** entire TCGA cohort; **(F)** GEO cohort.

ROC was utilized to evaluate the accuracy of the RS model in our training set. As shown in **Figure 3C**, the area under curve (AUC) for the model were 0.818, 0.776, and 0.771 for 1-, 3-, and 5- years, respectively. We used the median value of RS as the boundary to classify the samples into HRG and LRG. K-M survival analysis showed that LRG had a better prognosis than HRG (**Figure 4C**).

We performed survival analyses in the TCGA test set, all TCGA set, and the GEO cohort. HRG had a significantly worse prognosis than LRG (**Figure 4D–F**). In the three validation sets, the 1-year AUC was 0.761, 0.664, and 0.589, respectively (**Figures 3D–F**). In the GEO cohort, the low AUC values at 1, 3, and 5 years were partly due to the short median follow-up time of patients.

### Construction of a Nomogram Containing RS for TME-Related Genes

**Figure 5:**
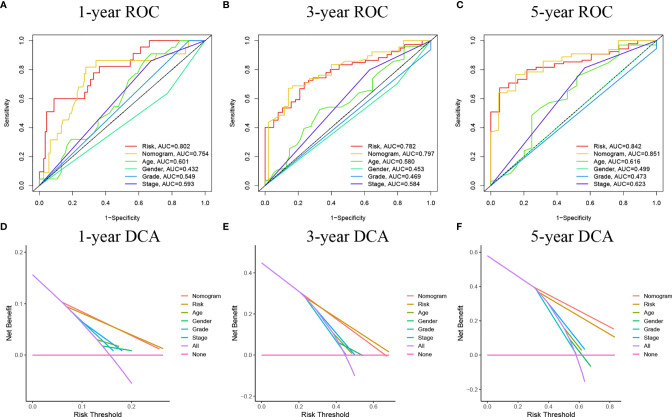
**(A–C)** ROC curves of the nomograms compared with other clinical variables. **(D–F)** Decision curve analysis (DCA) curves can evaluate the clinical benefit of the nomograms and their potential scope of application. None indicates that all samples are negative and none are treated, therefore the net benefit is zero. All indicates that all samples are positive and all are treated. The x-axis represents the threshold probability.

We used univariate and multivariate Cox regression to examine the relationship between potential variables and OS, which included RS, TNM stage, pathological grade, age, and sex (**Table 3**). The results showed that RS (HR: 1.045, 95% CI: 1.019–1.071, p<0.001), TNM stage(HR: 2.127, 95% CI: 1.416–3.196, p<0.001), and age (HR: 1.034, 95% CI: 1.017–1.052, p<0.001) were independent risk factors. Next, we constructed a nomogram including RS and clinical features (**Figure 4A**). Individualized 1-year, 3-year, and 5-year survival rates can be visualized based on the nomogram. For example, a 63-year-old male patient diagnosed with BLCA has clinical features of T3M0N0, high pathological grading, and low RS. The clinician can then calculate the corresponding scores for the variables, with a final total score of 442. Thus, the patient’s 1-year, 3-year, and 5-year survival rates can be inferred to be 0.944,0.797, and 0.685, respectively. The results showed a significant impact of RS on survival prediction. We drew calibration graphs of 1-year, 3-year, and 5-year OS in the training sets to demonstrate the consensus of our model with the real results (**Figure 4B**). The AUC of the model was higher than the other impact factors in the 3-year and 5-year ROC curve (**Figures 5B, C**). However, the RS was higher than the AUC of the nomogram at 1-year ROC curve, which was the highest of all factors (**Figure 5A**). Finally, DCA to assess the clinical utility of nomograms. Both nomogram and RS showed good consistency in forecasting OS at 1, 3, and 5 years compared with a singular prognostic factor (**Figures 5D–F**).

**Table 3:** Univariable and multivariable Cox regression to analyze the relationship between the RS and clinical prognosis.

Variables		Univariable Analysis			Multivariable Analysis	
	HR	95%*CI*	*P-*Value	HR	95%*CI*	*P-*Value
Age	1.038	1.021–1.056	<0.001	1.034	1.017–1.052	<0.001
Gender	0.891	0.632–1.257	0.511	~	~	~
Grade	1.779	0.438–7.227	0.421	~	~	~
Stage	2.330	1.555–3.492	<0.001	2.127	1.416–3.196	<0.001
Risk Score	1.057	1.032–1.083	<0.001	1.045	1.019–1.071	<0.001

### Comparison of the Eight-Gene Signature Risk Model With Other Models

**Figure 6:**
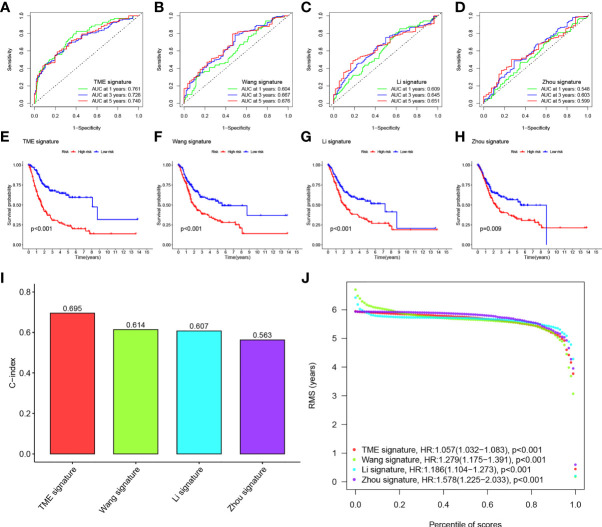
**(A–D)** ROC of three other published gene signatures. **(E–H)** KM curves of three other published gene signatures. **(I)** Concordance index (C-index) of the four prognostic risk models including our model, which has the highest C-index. **(J)** Restricted mean survival (RMS) time curve of all five prognostic risk models, revealing an overlap of 60 months.

We compared the prediction models of our eight-gene signature with other models to demonstrate the predictive performance of our model, including models of the identified 3-gene (25), 5-gene (26), and 7-gene signature (27). We also computed RS for every sample by multivariate Cox regression analysis. We evaluated the ROC of the four models according to the corresponding genes. The cases were then classified into HRG and LRG based on the median RS value. In all four models, there was a significant difference in survival time between patients in the HRG and LRG groups, with patients in LRH having a better prognosis than those in HRG (**Figures 6E–H**). However, the ROC of the previous models showed lower AUCs and, therefore, the other three models have worse predictions compared to our nomogram. (**Figures 6A–D**).

We calculated the C-index of the four models and found that our model has the highest C-index with 0.695 (**Figure 6I**). RMS time was used to estimate the forecasting effectiveness of the model at different time points. Our model was found to outperform the other two gene signatures at > 60 months except for Zhou signature. This demonstrates that our nomogram has an advantage over the other models in predicting both patient survival up to 5 years and patient survival beyond 60 months (**Figure 6J**).

### Functional Analysis of Genes Between HRG and LRG

**Figure 7:**
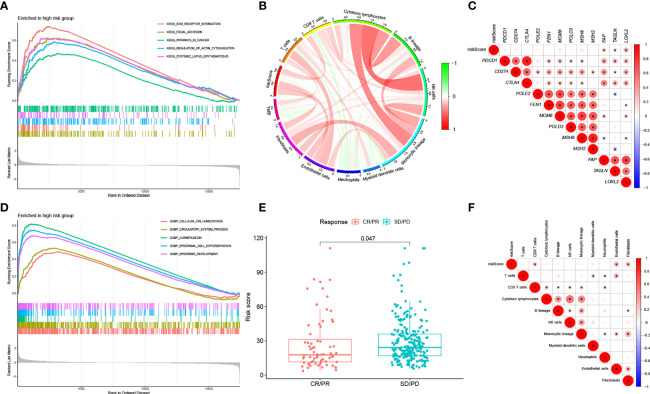
**(A)** KEGG analysis of our signature in the HRG. **(B)** Correlation between RS, immune cell score and TMB. **(C)** Correlation between RS of BLCA samples and expression of representative genes for generic pathway targets in oncology. **(D)** GO analysis of our signature in the HRG. **(E)** RS according to the effectiveness of immunotherapy expressed as complete response (CR), partial response (PR), stable disease (SD), or progressive disease (PD). **(F)** Correlation between RS of BLCA samples and immune scores.

We ran GSEA on samples within the HRG and LRG. We identified the following pathways with associated normalized enrichment score (NES) and the adjusted p-value (*q*-value) enriched in the HRG (**Figures 7A, D**): *KEGG_FOCAL_ADHESION* (NES =2.1496, *q* =1.57E-08), *KEGG_ECM_RECEPTOR_INTERACTION* (NES =2.2856, *q* =4.50E-07), *KEGG_REGULATION_OF_ACTIN_CYTOSKELETON* (NES =2.0206, *q* =1.25E-06), *KEGG_SYSTEMIC_LUPUS_ERYTHEMATOSUS* (NES =2.0084, *q* =4.96E-05), *KEGG_PATHWAYS_IN_CANCER* (NES =1.7137, *q* =0.0003), *GOBP_CELLULAR_ION_HOMEOSTASIS* (NES =1.9040, *q* =1.71E-08), *GOBP_CIRCULATORY_SYSTEM_PROCESS* (NES =2.0543, *q* =1.71E-08), *GOBP_CORNIFICATION* (NES =2.5951, *q* =1.71E-08), *GOBP_EPIDERMAL_CELL_DIFFERENTIATION* (NES =2.6868, *q* =1.71E-08), *GOBP_EPIDERMIS_DEVELOPMENT* (NES =2.5372, *q* =1.71E-08).

Classical pathways in tumors include immune checkpoints, DNA duplication, mismatch repair, and epithelial-mesenchymal transition (EMT). The correlation of RS with target genes was analyzed by extracting the expression of the relevant pathway target genes from the samples. The results showed that RS was positively correlated with EMT-related genes (*F-AP*, *TAGLN*, and *LOXL2*) (**Figure 7C**).

We computed the immune cell fraction for every sample of the TCGA-BLCA set using an MCP counter and then compared their correlation with RS. As shown in **Figure 7F**, RS was positively correlated with CD8+ T cells (p<0.05), endothelial cells (p<0.05), and fibroblasts (p<0.05). In addition, we evaluated the correlation between RS, TMB, and infiltrating cells by MCP counter with the results shown in **Figure 7B**.

**Figure 8:**
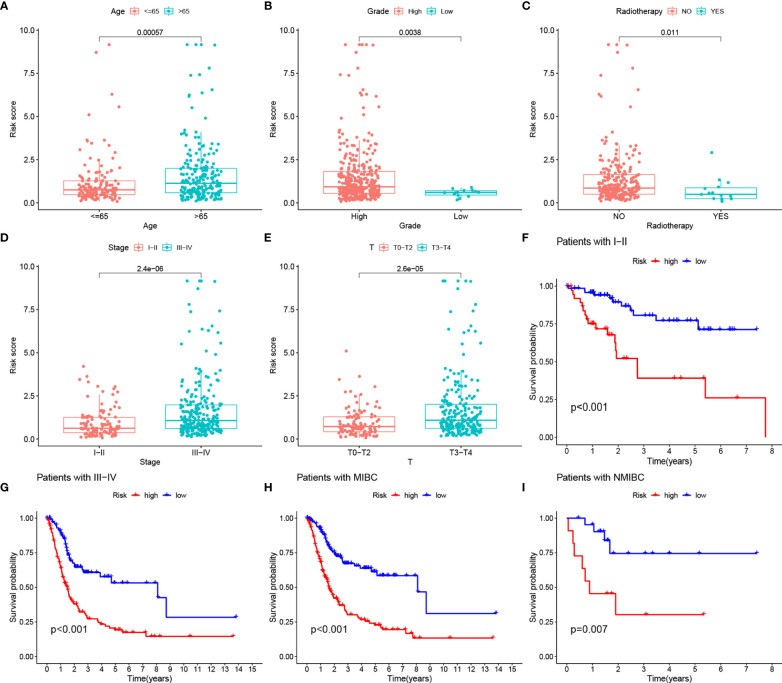
**(A–E)** RS comparison of clinical information of BLCA samples. **(F, G)** Evaluation of the eight-gene risk model in different stage groups with OS KM curves comparing HRG and LRG. **(H, I)** Evaluation of the eight-gene risk model in different degree of tumor infiltration groups with OS KM curves comparing HRG and LRG.

Our analysis showed that there were also differences in RS between clinical subgroups of BLCA patients. More specifically, patients older than 65 years, with no history of radiotherapy, female, and with high TNM stage had higher RS (**Figures 8A–E**). The K-M survival curves of patients in both stage I-II and III-IV subgroups also proved our results (**Figures 8F–H**). In addition, the K-M survival curves of patients in both MIBC and NMIBC subgroups also proved our results (**Figures 8H, I**).

### Prediction of Response to Immunotherapy Based on RS Model

We selected patients with urologic tumors in the IMvigor210 cohort who had received PD-L1 blockade therapy to observe the effectiveness against immunotherapy. We calculated the RS for each sample and classified them into HRG and LRG based on the median RS value. the RS showed significantly higher values (p = 0.047) in patients with SD/PD compared to patients with CR/PR (**Figure 7E**). This suggests that our RS model may be useful in predicting patient response to PD-L1 blocking therapy.

## Discussion

The major discovery of our work was a prediction model according to the TME - associated gene signature of BLCA. TME heterogeneity has an essential role in patient prognosis and in predicting the effectiveness of targeted therapies (28–30). 4061 TME-related genes and 359 BLCA samples of TCGA were evaluated in this work. We applied the NMF method to distinguish two molecular clusters, which is a promising novel clustering method. There were differences in immune scores for TME-infiltrating cell types between C1 and C2. In addition, correlation analysis with prognostic indicators OS and PFS also showed differences between C1 and C2. These results demonstrate the heterogeneity of TME.

We found that our prognostic model based on eight TME-related genes (*CABP4*, *ZNF432*, *BLOC1S3*, *CXCL11*, *ANO9*, *OAS1*, *FBN2*, *CEMIP*) was able to accurately forecast the probability of survival in individuals with BLCA. Prior researches have examined several genes included in our signature under a variety of tumors. For instance, *CXCL11* has an important role in the production of chemokines. First, these mediators can trigger the accumulation of CD8+ T cells that can contribute to the elimination of the tumor. Secondly, the production of these chemokines by tumor tissue may trigger the migration and activation of immune cells including myeloid-derived suppressor cells and regulatory T cells, which act in favor of the tumor and its progress (31). *FBN2* is highly expressed in lung cancer tissues, and as an oncogene, it affects the pathogenesis of lung cancer (32). In addition, Chen Y (33) found that *CEMIP* can promote a variety of tumor processes by affecting tumor proliferation, dedifferentiation, and the tumor microenvironment. In terms of molecular mechanisms, existing research has shown that *CEMIP* mainly affects the WNT and EGFR signaling pathways.

Multivariate Cox regression analysis revealed RS (HR =1.045, 95% CI: 1.019-1.071) as an independent risk factor, which was validated in both the internal and external sets. We then defined HRG and LRG in the TCGA cohort based on the median RS value. For example, we found that RS was positively correlated with EMT-related genes. In general, a greater degree of immune infiltration could explain a more immune defense in LRG, and thus a more favorable prognosis. Conversely, positively correlated EMT genes may lead to a higher propensity for metastasis and a poorer outcome for BLCA patients in HRG. In addition, RS differs between clinical subgroups of BLCA patients, with a worse prognosis in the elderly, women, and patients with high TNM staging. Thus, our results suggest that RS can provide resilient risk stratification for BLCA patients.

Nomograms are a well-recognized statistical method for visualizing and predicting the probability of survival of patients (34–36). We innovatively constructed an advanced nomogram for the prognosis of BLCA patients according to TME-RS. The ROC and DCA proved that they can exactly assess the outcome of patients. When we compared our nomogram with three other predictive genes signatures by Wang et al. (27), Zhou et al. (25), and Li et al. (26). Our nomogram showed the highest C-index. These findings suggest that the clinical utility of our constructed nomogram is outperformed by other models. Next, our team will transfer this RS model to other urological tumors such as renal clear cell carcinoma to demonstrate the potential pan-cancer usability of the model.

Of course, there were still some deficiencies in our research. First of all, all our data come from the TCGA database and GEO database, and the sample size was not large enough, which may lead to the bias of the results. Secondly, our conclusions were also lack experimental verification. Therefore, it was necessary to conduct multicenter, large sample, prospective double-blind trials for further verification in the future.

## Conclusions

In conclusion, our eight TME-associated gene signatures combined with clinical features may more accurately predict patient survival at 1, 3, and 5 years. Both external and internal validation were able to have the ability to verify the forecasting capability of the model. RS can be promising to predict the response to immunotherapy of BLCA individuals with PD-L1 blocking therapy.

## Data Availability Statement

The datasets presented in this study can be found in online repositories. The names of the repository/repositories and accession number(s) can be found in the article/**Supplementary Material**.

## Author Contributions

CX, ZK, and DP designed this work. YL analyzed the data. CX and DP wrote this manuscript. ZK edited and revised the manuscript. JG, NL and YY did a lot of work in revising the paper, including re-screening the data, analyzing the data, plotting the figures and the layout of the figures. YY embellished the language of the paper. All authors approved this manuscript.

## Funding

This study was funded by the Health Care Commission of Henan Province (201403125, LHGJ20190422) and the Department of Education of Henan Province (21A320037).

## Conflict of Interest

The authors declare that the research was conducted in the absence of any commercial or financial relationships that could be construed as a potential conflict of interest.

## Publisher’s Note

All claims expressed in this article are solely those of the authors and do not necessarily represent those of their affiliated organizations, or those of the publisher, the editors and the reviewers. Any product that may be evaluated in this article, or claim that may be made by its manufacturer, is not guaranteed or endorsed by the publisher.

## Abbreviations

TME, Tumor microenvironment; BLCA, bladder urothelial carcinoma; NMF, non-negative matrix factorization; K-M, Kaplan-Meier; ROC, receiver operating characteristic; DCA, decision curve analysis; RS, Risk score; HRG, high-risk group; LRG, low-risk group; MIBC, muscle-invasive bladder cancer; UICC, Union International Center of Cancer; OS, overall survival; PFS, Progression-free survival; GEO, Gene-Expression Omnibus; TCGA-BLCA, The Cancer Genome Atlas-Bladder Cancer; CNV, copy number variation; RSS, sum of squared residuals; MCP, Microenvironment Cell Populations; TMB, tumor mutation burden; RMS, restricted mean survival; CR, complete response; PR, partial response; SD, stable disease; PD, progressive disease; GSEA, Gene set enrichment analysis; PD-L1, programmed death ligand 1; HR, hazard ratio; NES, normalized enrichment score; EMT, epithelial-mesenchymal transition.

## References

[1] Zhang M Zhang X Yu M Zhang W Zhang D Zeng S. A Novel Ferroptosis-Related Gene Model for Overall Survival Predictions of Bladder Urothelial Carcinoma Patients. Front Oncol (2021) 11:698856. doi: 10.3389/fonc.2021.698856 34386423 PMC8353278

[2] Bray F Ferlay J Soerjomataram I Siegel R Torre L Jemal A. Global Cancer Statistics 2018: GLOBOCAN Estimates of Incidence and Mortality Worldwide for 36 Cancers in 185 Countries. CA: Cancer J Clin (2018) 68:394–424. doi: 10.3322/caac.21492 30207593

[3] Witjes J Bruins H Cathomas R Compérat E Cowan N Gakis G. European Association of Urology Guidelines on Muscle-Invasive and Metastatic Bladder Cancer: Summary of the 2020 Guidelines. Eur Urol (2021) 79:82–104. doi: 10.1016/j.eururo.2020.03.055 32360052

[4] van den Bosch S Alfred Witjes J. Long-Term Cancer-Specific Survival in Patients With High-Risk, Non-Muscle-Invasive Bladder Cancer and Tumour Progression: A Systematic Review. Eur Urol (2011) 60:493–500. doi: 10.1016/j.eururo.2011.05.045 21664041

[5] Magers M Lopez-Beltran A Montironi R Williamson S Kaimakliotis H Cheng LJH. Staging of Bladder Cancer. Histopathology (2019) 74:112–34. doi: 10.1111/his.13734 30565300

[6] Barton M. High Morbidity and Mortality Found for High-Risk, Non-Muscle-Invasive Bladder Cancer. CA: Cancer J Clin (2013) 63:371–2. doi: 10.3322/caac.21201 24122724

[7] Chedgy E Black P. Radical Cystectomy and the Multidisciplinary Management of Muscle-Invasive Bladder Cancer. JAMA Oncol (2016) 2:855–6. doi: 10.1001/jamaoncol.2016.0149 27149036

[8] Jain P Kathuria H Momin M. Clinical Therapies and Nano Drug Delivery Systems for Urinary Bladder Cancer. Biomed Pharmacother (2021) 226:107871. doi: 10.1016/j.pharmthera.2021.107871 33915179

[9] Liang Y Ye F Xu C Zou L Hu Y Hu J. A Novel Survival Model Based on a Ferroptosis-Related Gene Signature for Predicting Overall Survival in Bladder Cancer. Front Oncol (2021) 21:943. doi: 10.1186/s12885-021-08687-7 PMC8380338 34418989

[10] Cao R Ma B Wang G Xiong Y Tian Y Yuan L. Identification of Autophagy-Related Genes Signature Predicts Chemotherapeutic and Immunotherapeutic Efficiency in Bladder Cancer (BLCA). J Cell Mol Med (2021) 25:5417–33. doi: 10.1111/jcmm.16552 PMC8184684 33960661

[11] Chen F Wang Q Zhou Y. The Construction and Validation of an RNA Binding Protein-Related Prognostic Model for Bladder Cancer. BMC Cancer (2021) 21:244. doi: 10.1186/s12885-021-07930-5 33685397 PMC7938493

[12] Ren F Zhao Q Zhao M Zhu S Liu B Bukhari I. Immune Infiltration Profiling in Gastric Cancer and Their Clinical Implications. Cancer Sci (2021) 112(9):3569–84. doi: 10.1111/cas.15057 PMC8409427 34251747

[13] Hui L Chen Y. Tumor Microenvironment: Sanctuary of the Devil. Cancer Lett (2015) 368:7–13. doi: 10.1016/j.canlet.2015.07.039 26276713

[14] Whiteside TJO. The Tumor Microenvironment and Its Role in Promoting Tumor Growth. Oncogene (2008) 27:5904–12. doi: 10.1038/onc.2008.271 PMC3689267 18836471

[15] Mariathasan S Turley S Nickles D Castiglioni A Yuen K Wang Y. Tgfβ Attenuates Tumour Response to PD-L1 Blockade by Contributing to Exclusion of T Cells. Nature (2018) 554:544–8. doi: 10.1038/nature25501 PMC6028240 29443960

[16] Wang Y Chen L Yu M Fang Y Qian K Wang G. Immune-Related Signature Predicts the Prognosis and Immunotherapy Benefit in Bladder Cancer. Cancer Med (2020) 9:7729–41. doi: 10.1002/cam4.3400 PMC7571842 32841548

[17] Aran D Hu Z Butte AJ. Xcell: Digitally Portraying the Tissue Cellular Heterogeneity Landscape. Genome Biol (2017) 18:220. doi: 10.1186/s13059-017-1349-1 29141660 PMC5688663

[18] Becht E Giraldo NA Lacroix L Buttard B Elarouci N Petitprez F. Estimating the Population Abundance of Tissue-Infiltrating Immune and Stromal Cell Populations Using Gene Expression. Genome Biol (2016) 17:218. doi: 10.1186/s13059-016-1070-5 27765066 PMC5073889

[19] Chifman J Pullikuth A Chou JW Bedognetti D Miller LD. Conservation of Immune Gene Signatures in Solid Tumors and Prognostic Implications. BMC Cancer (2016) 16:911. doi: 10.1186/s12885-016-2948-z 27871313 PMC5118876

[20] Li B Severson E Pignon J-C Zhao H Li T Novak J. Comprehensive Analyses of Tumor Immunity: Implications for Cancer Immunotherapy. Genome Biol (2016) 17:174. doi: 10.1186/s13059-016-1028-7 27549193 PMC4993001

[21] Newman AM Liu CL Green MR Gentles AJ Feng W Xu Y. Robust Enumeration of Cell Subsets From Tissue Expression Profiles. Nat Methods (2015) 12:453–7. doi: 10.1038/nmeth.3337 PMC4739640 25822800

[22] Rooney M Shukla S Wu C Getz G Hacohen NJC. Molecular and Genetic Properties of Tumors Associated With Local Immune Cytolytic Activity. Cell (2015) 160:48–61. doi: 10.1016/j.cell.2014.12.033 25594174 PMC4856474

[23] Tirosh I Izar B Prakadan SM Wadsworth MH Treacy D Trombetta JJ. Dissecting the Multicellular Ecosystem of Metastatic Melanoma by Single-Cell RNA-Seq. Science (2016) 352:189–96. doi: 10.1126/science.aad0501 PMC4944528 27124452

[24] Gaujoux R Seoighe C. A Flexible R Package for Nonnegative Matrix Factorization. BMC Bioinf (2010) 11:367. doi: 10.1186/1471-2105-11-367 PMC2912887 20598126

[25] Li F Teng H Liu M Liu B Zhang D Xu Z. Prognostic Value of Immune-Related Genes in the Tumor Microenvironment of Bladder Cancer. Front Oncol (2020) 10:1302. doi: 10.3389/fonc.2020.01302 32850407 PMC7399341

[26] Li X Feng J Sun Y Li X. An Exploration of the Tumor Microenvironment Identified a Novel Five-Gene Model for Predicting Outcomes in Bladder Cancer. Front Oncol (2021) 11:642527. doi: 10.3389/fonc.2021.642527 34012914 PMC8126988

[27] Wang Z Tu L Chen M Tong S. Identification of a Tumor Microenvironment-Related Seven-Gene Signature for Predicting Prognosis in Bladder Cancer. BMC Cancer (2021) 21:692. doi: 10.1186/s12885-021-08447-7 34112144 PMC8194149

[28] Hanahan D Coussens L. Accessories to the Crime: Functions of Cells Recruited to the Tumor Microenvironment. Cancer Cell (2012) 21:309–22. doi: 10.1016/j.ccr.2012.02.022 22439926

[29] Hanahan D Weinberg RJC. Hallmarks of Cancer: The Next Generation. Cell (2011) 144:646–74. doi: 10.1016/j.cell.2011.02.013 21376230

[30] Schulz M Salamero-Boix A Niesel K Alekseeva T Sevenich L. Microenvironmental Regulation of Tumor Progression and Therapeutic Response in Brain Metastasis. Front Immunol (2019) 10:1713. doi: 10.3389/fimmu.2019.01713 31396225 PMC6667643

[31] Nazari A Ahmadi Z Hassanshahi G Abbasifard M Taghipour Z Falahati-Pour SK. Effective Treatments for Bladder Cancer Affecting CXCL9/CXCL10/CXCL11/CXCR3 Axis: A Review. Oman Med J (2020) 35:e103. doi: 10.5001/omj.2020.21 32181005 PMC7064791

[32] Hong Q Li R Zhang Y Gu KJC m. biology. Fibrillin 2 Gene Knockdown Inhibits Invasion and Migration of Lung Cancer Cells. Cell Mol Biol (2020) 66:190–6. doi: 10.14715/cmb/2020.66.7.29 33287941

[33] Chen Y Zhou H Jiang W Wang J Tian Y Jiang Y. The Role of CEMIP in Tumors: An Update Based on Cellular and Molecular Insights. Biomed Pharmacother (2021) 146:112504. doi: 10.1016/j.biopha.2021.112504 34922110

[34] Fakhry C Zhang Q Nguyen-Tân P Rosenthal D Weber R Lambert L. Development and Validation of Nomograms Predictive of Overall and Progression-Free Survival in Patients With Oropharyngeal Cancer. J Clin Oncol (2017) 35:4057–65. doi: 10.1200/JCO.2016.72.0748 PMC5736236 28777690

[35] Previs R Bevis K Huh W Tillmanns T Perry L Moore K. A Prognostic Nomogram to Predict Overall Survival in Women With Recurrent Ovarian Cancer Treated With Bevacizumab and Chemotherapy. Gynecol Oncol (2014) 132:531–6. doi: 10.1016/j.ygyno.2014.01.036 24472410

[36] Wang S Fuller C Kim J Sittig D Thomas C Ravdin P. Prediction Model for Estimating the Survival Benefit of Adjuvant Radiotherapy for Gallbladder Cancer. J Clin Oncol (2008) 26:2112–7. doi: 10.1200/JCO.2007.14.7934 18378567

